# Exploring hospital efficiency within and between Italian regions: new empirical evidence

**DOI:** 10.1007/s11123-022-00633-4

**Published:** 2022-03-11

**Authors:** Cristian Barra, Raffaele Lagravinese, Roberto Zotti

**Affiliations:** 1 Department of Economics and Statistics, University of Salerno, Fisciano, SA Italy; 2 Department of Economics and Finance, University of Bari “A.Moro”, Bari, BA Italy; 3 Department of Economics and Statistics “Cognetti de Martiis”, University of Torino, Torino, TO Italy

**Keywords:** I14, I18, C67, Benefit of Doubt, MetaFrontier, Health Efficiency, Regional Inequality

## Abstract

This paper investigates the efficiency of Italian hospitals and how their performances have changed over the years 2007–2016, characterized by the great economic recession and budget constraints. We apply the Benefit of Doubt (BoD) approach to determine a composite index that considers the multi-dimensionality of the hospital outcome to be used as main output in a metafrontier production function based on a stochastic frontier framework. The efficiency score distribution is then used to construct a Theil index in order to compare, over time, the inequality of the estimated efficiency between hospitals, both within and between regions. The main findings show that the primary source of inefficiency comes from managerial inefficiency especially for hospitals located in southern regions. A clear and persistent North-South gap in efficiency performances of hospitals has been found along with an increase in the inequality in terms of efficiency between the areas of the country mostly determined by between region inequality.

## Introduction

The Italian National Health Service (NHS), introduced in 1978, is a universal health care system providing comprehensive health insurance coverage and uniform health benefits to the whole population. It is inspired by the two fundamental pillars enshrined in Article 32 of the Italian Constitution: universality and equality. While the principle of universality has never been questioned, the principle of equality, especially if broken down into terms of equality of services in the local territories, has been repeatedly overlooked in recent years. The last decade has been characterized by the profound financial crisis, exacerbating the economic differences between North and South (see about this aspect Lagravinese (2015)) with inevitable effects also on the demand and supply of health. Indeed, inequalities have started to increase again with evident effects on available healthcare resources, on the quality of uneven healthcare services, and on the living conditions of citizens.

Since its introduction, and as in other European countries (see Costa-Font and Greer (2013)), the Italian NHS has undergone important reforms to decentralize health management and policy responsibilities to the sub-layers of government (Turati, 2013). As a result of a federal reform (Legislative Decree 56/2000), each region is in charge of organizing the health system, following the general guidelines defined by the central government that is responsible for setting the Essential Levels of Health Services (LEA, *Livelli Essenziali di Assistenza*).^1^ However, the separation of financing from expenditure responsibilities in the provision of LEA, and before LEA in the provision of uniform levels of service, has provided a non-negligible incentive to the uncontrolled growth of Italian health expenditures and has historically contributed to creating bailing out expectations in regional behavior (Liberati, 2003), especially in a context of often inadequate regional health governance and accountability (Carinci et al., 2012).

The public policies concerning a reduction in beds, in medical and nursing staff, hospital mergers and acquisitions as well as lower investments in infrastructure are now subject to revaluation in many countries. Even the decentralized organization itself is questioned and, in many cases, possible scenarios of re-centralization of the health system are well thought-out (Mosca, 2007). In this respect, Italy is among the western countries that have significantly reduced healthcare spending by reshaping its hospital organization and decentralizing management at regional level. The effects of these policies are still controversial to this day, but what is undoubtedly an established fact is that the inequalities between the regional systems in these 20 years have not diminished. Furthermore, the recovery plans imposed by the central government in 2007 for the regions with high deficits^2^ have certainly favored the reduction of healthcare expenditures and have proved to be an effective mechanism for eliminating sub-national governments deficits (Bordignon et al., 2020). Nevertheless, there is still no clear consensus in the literature whether these plans, and in general the decentralization process, have also had an impact on increasing health inequalities between or within regions. Our work attempts to enrich this literature looking at how hospital efficiency (one of the pillar of health services restructuring by the regions) has changed over the last decade within and across regions.

The economic literature has so far produced a rich and consolidated series of works to study hospital efficiency. As far as Italy is concerned, most of them have investigated if the efficiency has changed either with the introduction of new accounting systems, such as the use of the diagnostic-related groups (Barbetta et al., 2007, Cavalieri et al., 2018), or due to the ownership of hospitals (Cellini et al., 2000, Colombi et al., 2017, Daidone and D’Amico, 2009). To the best of our knowledge, there are no studies that measure in a more holistic way how efficiency has changed over the years and whether the regional policies have actually reduced the differences in hospitals between and within regions. This paper attempts to fill this gap. In particular, the aim of the present study is threefold.

The first contribution of the paper regards the methodology used to measure the efficiency of hospitals. Indeed, health performances are usually averaged across several dimensions to obtain a composite indicator useful for ranking and comparisons. However, the obtained average score can hide contrasting attitudes and specialisations, being a crucial issue the definition of a proper set of weights to aggregate different subjects.^3^ We apply the benefit of doubt (BoD) approach to determine a composite index that considers the multi-dimensionality of the hospital outcome. As far as we know, the BoD has received scarce attention so far in the health care sector. Nonetheless, in the absence of information about value judgments, as it is the case of hospitals, the BoD methodology can be an useful tool to account for the complexity of the health care system (Matos et al., 2021). The BoD aggregates linearly quantitative performance sub-indicators into a single composite one using the combination of weights that is the most convenient for the evaluated hospital (Cherchye et al., 2007). This is done by implicitly assigning less (more) weight to those sub-indicators or aspects of performance that the particular hospital is relatively weak (strong) when compared with all others in the sample (Karagiannis and Paschalidou, 2017).^4^ The high flexibility in terms of exogenous assumptions for setting weights, makes the BoD an appropriate approach to account for the specializations of individual hospitals.

Secondly, the composite indicator generated by the BoD is then used as output in a metafrontier production function based on a stochastic frontier framework.^5^ More specifically, we apply a two-step stochastic frontier approach recently developed by Huang et al. (2014), based on the idea that hospitals have access, potentially, to distinct production technologies in different time periods, being conditioned by several factors such as, for instance, environmental characteristics, regulation and the availability of resources. The analysis is carried out using hospital data over almost one decade: in 2007, the year before the great recession and when, with the introduction of the recovery plans, a number of regions had to substantially reconsider their offer on the territory and in 2016, the last year for which the data are available in detail for all Italian healthcare facilities. To account for the change in efficiency between the two years, the metafrontier has been used. To the best of our knowledge, this is the first work that applies this methodology to the Italian hospital context. The main findings show that the primary source of inefficiency comes from managerial inefficiency especially for hospitals located in southern regions.

The final contribution of the paper is an attempt to link the literature on the hospitals’ efficiency and inequality in the healthcare services at local level. The efficiency score distribution is employed to compute the Theil index (Theil, 1967), a perfect decomposable inequality index, to compare over time the inequality of the estimated efficiency between hospitals in the same region and between different regions. The results of this analysis allow us to make some more general considerations regarding the evolution of the healthcare services in Italy. The findings show a clear and persistent North-South gap in efficiency performances of hospitals along with an increase in the inequality in terms of efficiency between the areas of the country mostly determined by between region inequality. The empirical evidence provides helpful recommendations for policy makers regarding whether local level provision of hospital services need to be improved or whether the regional policies implemented in these years must be reconsidered in order to favor a convergence process.

The rest of the paper is organized as follows. The next section describes the methodology employed, Section 3 presents the data and provides some descriptive statistics, Section 4 discusses the results both in the metafrontier and inequality analyses and finally, Section 5 concludes and offers some policy implications.

## Methodology

### Efficiency of hospitals

We calculate the hospital’s relative efficiency at converting inputs into a production set while maximizing outputs. In the literature of productive efficiency analysis and frontier estimation, hospital efficiency has been estimated by two main alternative methods such as data envelopment analysis - DEA (Charnes et al., 1978, Farrell, 1957) and stochastic frontier analysis—SFA (Aigner et al., 1977, Meeusen and van den Broeck, 1977). The main advantage of the former method is the non-parametric treatment of the frontier that does not require the underlying production function to belong to any specific functional form.^6^ Although its underlying assumptions provide a way to economize on data requirements, making efficiency analysis possible even when other methods of estimation would not be applicable (Patrizii and Resce, 2013), it ignores any stochastic noise in the data. The advantage of the SFA, instead, is in its stochastic treatment of these deviations.^7^ However, the data used in these analyses may be relatively noisy, so that the deterministic approach (such as the DEA) seems to be inappropriate. Moreover, the units analyzed may apply different production technologies making also the parametric approach (such as the SFA) inappropriate. To incorporate the benefits of both approaches and to bridge the gap between SFA and DEA, several solutions have been proposed in the literature. For a recent and comprehensive view of the theoretical and empirical methods approaches to productivity and efficiency analysis, see Sickles and Zelenyuk (2019).

To comply with the characteristics of the hospital sector and to better understand the differences in average health costs and productivity among hospitals operating in the same environment, more standard techniques along with alternative approaches are used. An extension of the stochastic frontier is applied to estimate the determinants of persistent (long-run) and transient (short-run) inefficiencies (Colombi et al., 2017). With the aim of measuring the impact of environmental characteristics on hospitals’ technical efficiency, the conditional-efficiency approach (Cordero et al., 2015, Halkos and Tzeremes, 2011, Mastromarco et al., 2019, Varabyova et al., 2017) as well as a two-stage semiparametric bootstrap-based approach are applied (Cavalieri et al., 2018, Chowdhury and Zelenyuk, 2016). The importance of the spatial dependent heterogeneity in hospital technical efficiency is also underlined, through a spatial stochastic frontier, to separate spatial issues from the effects of geographical institutional factors (Auteri et al., 2019, Cavalieri et al., 2020). Stochastic input and output distance functions are also employed (Daidone and D’Amico, 2009, Jiang and Andrews, 2020) to model multiple input and output technologies. However, although extensive, the literature has not reached a clear consensus on which method should be adopted to measure the efficiency of healthcare provision institutions. For a comprehensive view, see Jacobs et al. (2009).

In our opinion, the stochastic frontier model is particularly well-suitable for the analysis of health facilities. It allows for stochastic errors, results cannot be severely affected by the presence of outliers, statistical inferences can be drawn, and the estimated parameters (which significance can be tested) can provide potentially useful information in terms of policy implications. Moreover, since operating in more advantageous (more disadvantageous) environments may increase (decrease) a hospital’s efficiency, such factors should be taken into account in the efficiency estimation. We recognize merits of the SFA in integrating the environmental factors in a one-step approach avoiding collinearity issues between the error term and the efficiency scores. Potential limits in handling a multi-output and multi-input environment are solved through the use of a single composite outcome (BoD approach).

### The stochastic frontier analysis

To estimate hospital technical efficiency, we rely on a parametric approach, such as the SFA, through which a frontier is estimated on the relation between inputs and outputs. This can be, for example, a linear function, a quadratic function or a translog function. We use a more general functional form, that is the transcendental logarithmic or “translog” (see Barbetta et al. (2007), Colombi et al. (2017)). It may be preferred to the Cobb-Douglas form to overcome the latter restrictive elasticity of substitution and scale properties, and to allow for non-linear causalities. This method has become a common technique to assess production and inefficiencies in the production of good and services in several contexts, such as economics, health, education, and energy sectors. The methodology employed in this paper is developed by Huang et al. (2014). In a general canonical form, the stochastic frontier model is described by the following set of equations:

1 $${Y}_{it}={x}_{it}^{\prime}\beta +{\epsilon }_{it}$$

2 $${\epsilon }_{it}={\nu }_{it}-{u}_{it}$$

3 $${\nu }_{it} \sim N(0,{\sigma }_{\nu }^{2})$$

4 $${u}_{it} \sim {N}^{+}(0,{\sigma }_{u}^{2})$$

where *Y* denotes the output of the *i*_*th*_ hospital at time *t*, *x*_*i*_ is 1 × *k* vector of input of the *i*_*th*_ hospital at time *t*, *β* is *k* × 1 vector of unknown parameters to be estimated, *ϵ*_*it*_ is the random error, with *u*_*it*_ denoting the short-term inefficiency distributed by each unit as half normal, and *ν*_*it*_ is the stochastic component, distributed independently and identically as $N(0,{\sigma }_{\nu }^{2})$.

The model described in the system of Eqs. (1–4) is estimated by means of maximum-likelihood methods.^8^ The technical efficiency is calculated as the ratio of the observed output and the maximum feasible output on the production frontier. Formally:

5 $$T{E}_{it}=\frac{{Y}_{it}}{\hat{{Y}_{it}}}=\frac{{x}_{it}^{\prime}\beta +{\epsilon }_{it}}{{x}_{it}^{\prime}\beta +{\nu }_{it}}$$

where ${\hat{Y}}_{it}$ is the maximum feasible output that lies on the production frontier.

### Metafrontier model

We follow the procedure proposed by Huang et al. (2014), apply a two-stage parametric approach and estimate a metafrontier production function based on a stochastic frontier framework. We calculate technical efficiency scores for hospitals in separate groups adopting different technologies as we expect hospitals in 2007 and in 2016 not generated from a single production frontier. This procedure allows us to assess both the group-specific frontiers (hospitals in 2007 and hospitals in 2016) and the metafrontier (for both periods).

As in Battese et al. (2004) and O’Donnell et al. (2008), the comparable technical efficiency measures can be decomposed in technical efficiency scores specific to the two groups and technology gap ratios. The main difference between the method proposed by Huang et al. (2014) is that the latter’s second-step estimation of the metafrontier is still based on the stochastic frontier framework, rather than on a mathematical programming technique. Indeed, the two-step method applied by Battese et al. (2004) and O’Donnell et al. (2008) combines the stochastic frontier regression (used in the first step) to estimate the frontier of the separate groups with mathematical programming technique (in the second step) to obtain the metafrontier.^9^ The second-step estimation proposed by Huang et al. (2014) is still based on the stochastic frontier configuration, such that the metafrontier estimation is a stochastic metafrontier (SMF) regression method, with some important computational advantages. First, the usual statistical inferences can be performed without relying on simulations or bootstrap methods, as opposed to mathematical programming techniques.^10^ Second, the metafrontier makes possible to directly estimate the technology gaps by treating them as a conventional one-sided error term. This strategy allows us to separate the random shocks from the technology gaps.

The procedure is based on two stages. In the first stage, the group-specific frontiers (i.e. hospitals in 2007 and 2016) are estimated by means of a stochastic frontier model as in the system of Eqs. (1–4) for each hospital. The estimated parameter *β* associated to the pooled stochastic frontier model changes to *β*_*j*_ for each group *j*. In the second stage, a metafrontier is enveloped over the frontiers associated to each year (see for more details about the metafrontier optimization (O’Donnell et al., 2008)).

We calculate the technical efficiency (TE) associated to the metafrontier in order to compare the efficiency scores of the hospitals across different technology sets (frontiers). Formally:

6 $$T{E}_{it(j)}^{* }=\frac{{Y}_{it(j)}}{{Y}_{it}^{* }}=\frac{{x}_{it(j)}^{\prime}{\beta }_{j}+{\epsilon }_{it(j)}}{{x}_{it}^{\prime}{\beta }^{* }}$$

where ${Y}_{it}^{* }$ denotes the output on the metafrontier, β^*^ defines the parameters associated to the metafrontier function for all groups *j*, while $T{E}_{it(j)}^{* }$ represents the ratio of the observed output of hospital *i* in group *j* to the metafrontier output over time *t*.

The technology gap ratio ratio (TGR), described by the ratio of the output of the production function for hospital *i* over time *t* relative to the potential output of the metafrontier for a given set of input variables, is as follows:

7 $$TG{R}_{it(j)}=\frac{T{E}_{it(j)}^{* }}{T{E}_{it(j)}}$$

In particular, a technology gap exists due to the chosen technology depending on both economic and non-economic production environments. More specifically, TGR captures the difference between the productivity of the group and the metatechnology (i.e. the technology available to all hospitals) (see for more details the geographical representation associated to metafrontier in O’Donnell et al. (2008)).

Finally, the two previous terms can be also used to calculate the hospital technical efficiency with respect to the metafrontier production technology as opposed to the hospital’s technical efficiency with respect to the group-*j* production technology (MTE), as follows:

8 $$MT{E}_{it(j)}=TG{R}_{it(j)}\cdot T{E}_{it(j)}$$

### Inequality in efficiencies scores over time

In order to investigate inequality in terms of efficiency scores, we use the results of MTE. The distribution in the two periods is then computed disentangling between and within regions inequality using the Theil index (Theil, 1967), which is a perfectly decomposable inequality index, as follows:

9 $$T{I}_{it}=\mathop{\sum}\limits_{it}{f}_{it}\left(\frac{{\tau }_{it}}{\mu }\right)log\left(\frac{{\tau }_{it}}{\mu }\right)$$

where *f*_*it*_ is the group of hospital *i* at time *t*, *τ*_*it*_ is the efficiency index of the hospital *i* at time *t* and *μ* is the average of efficiency scores obtained from the full sample.

The index in Eq. (9) can be decomposed into a between and within group component as follows:

10 $$T{I}_{it}=\left[\mathop{\sum}\limits_{jt}{g}_{jt}\left(\frac{{\mu }_{jt}}{\mu }\right)log\left(\frac{{\mu }_{jt}}{\mu }\right)\right]+\mathop{\sum}\limits_{jt}T{I}_{jt}{g}_{jt}\left(\frac{{\mu }_{jt}}{\mu }\right)$$

where *j* refers to the sub-group, *g*_*jt*_ is the share of group *j* and *TI*_*jt*_ is the inequality in group *j* at time *t*. The between component of inequality is captured by the first term, i.e., the level of inequality if everyone within each group *j* had efficiency level *μ*_*j*_ at time *t*, the second term gives the within component of inequality (Cowell, 2000, Elbers et al., 2005).

## Data

### Inputs and outputs

The data were collected by the Ministry of Health. The dataset for 2016 can be consulted directly by downloading it from the government website whereas for the 2007 data, the Archive Internet Wayback Machine on the Ministry of Health website was used.

Data include information on different inputs and outputs usually considered in the studies on hospital efficiency (see, for instance, Barbetta et al. (2007), Cavalieri et al. (2018), Colombi et al. (2017), Jiang and Andrews (2020), Mastromarco et al. (2019)) with regard to 403 hospitals followed in two years (more specifically, we collect data on 29 accredited, 42 private and 332 public hospitals).

More specifically, five measures of inputs are included in the model. The first three measures reflect the number of personnel units: the number of physicians (*Physicians*), the number of nurses (*Nurses*) and the number of other personnel (*Other*). The fourth input refers to a measure of capital such as the number of available beds (*Beds*). The fifth input is the number of magnetic resonance imaging scans (*MRI scans*)^11^ per one thousand population at province level as a medical input to acknowledge the growth and importance of healthcare technology.^12^ With regard to the output side, three measures of outputs are included in the model: the number of discharged patients (*Discharged Patients*), the number of inpatient days (*Inpatient Days)*, and the number of emergency room treatments (*Emergency Room Treatments*).

Weighting outputs according to case-mix has been acknowledged as vital, particularly when the sample consists of hospitals of different sizes, or university hospitals together with other acute hospitals, to minimize intra-hospital as well as inter-hospital differences and therefore account for the different cost of hospital services (Chowdhury and Zelenyuk, 2016, Chowdhury et al., 2014, Mastromarco et al., 2019). To take heed of the complexity and different characteristics of the health services, following Colombi et al. (2017), we weight the three outputs using a weighted annual output of hospital *i* in year *t*, using the diagnosis-related group weight.

### Variability of (in)efficiency

It seems inappropriate to assume that efficiency will vary for each hospital in the same way. Institutions can react in very different ways to the contexts they operate in. To take into account the effects of factors that affect the performance of hospitals, we include a vector of exogenous variables in the variance of the inefficiency term (see Colombi et al. (2017)).

First of all, we reckon with the impact of ownership on hospital performances. In general, private ownership characterized by the presence of residual claimants should represent a powerful incentive to economic efficiency and cost reduction; on the contrary, public ownership and/or the absence of any claimant of residual earnings may induce shirking and could decrease effort, consequently reducing efficiency (Barbetta et al., 2007). We use a dummy variable taking the value of 1 in case the hospital has a private/accredited private health care (*Private*) ownership, and 0 otherwise.

Second, we also deal with the fact that, in some Italian regions, region-specific recovery plans, called “*Piani di Rientro*”, have been implemented since 2007 in order to recover from the budget deficit related to health care expenditures.^13^ Although part of the literature agrees on the fact that being included in such plans helps to contained costs (Atella et al., 2019), the consequences in terms of health outcomes are less clear.^14^ A dummy variable is therefore included: 1 if the hospital is located in a region included in the recovery plan (*Recovery Plans*), and 0 otherwise.

Finally, a macro-area dummy is also included to control for geographical area effects (*North*) taking the value of 1 in case the hospital is located in a region in the North of Italy, and 0 otherwise (Centre-South as reference group).

**Fig. 1:**
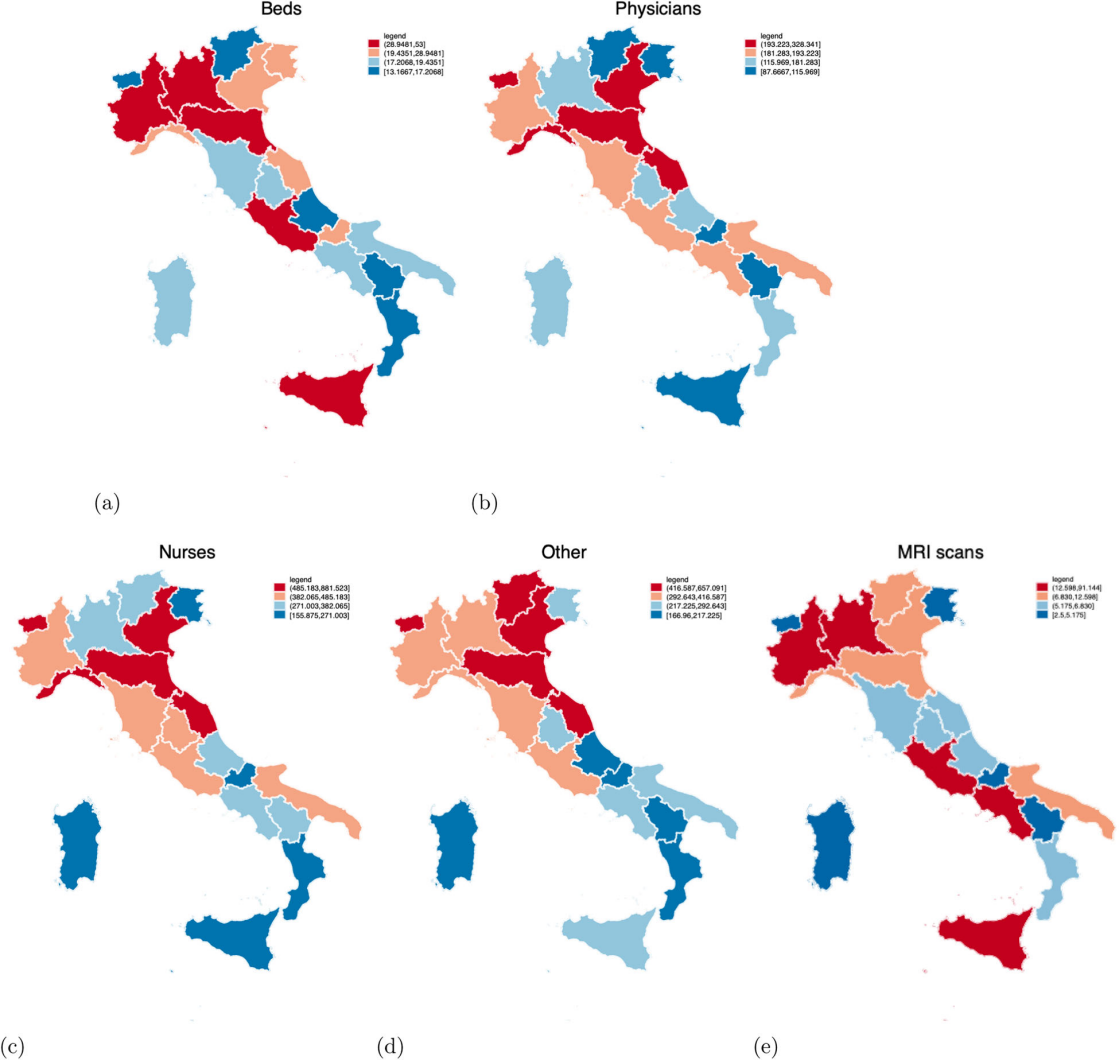
Descriptive statistics: Inputs (geographical variation). **a**–**e** The five measures of inputs included in the model such as the number of available beds (Beds), physicians (Physicians), nurses (Nurses), other personnel (Other), and magnetic resonance imaging scans (MRI scans), respectively

**Fig. 2:**
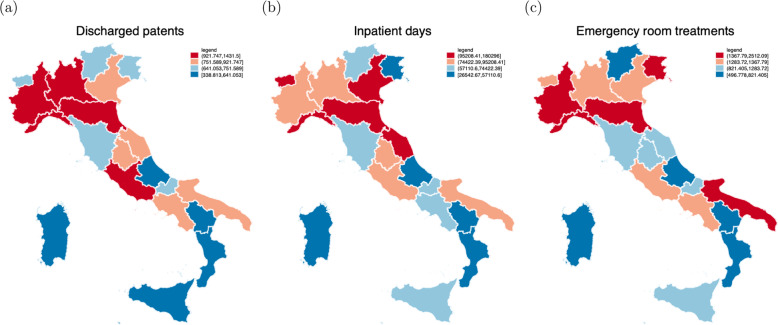
Descriptive statistics: Outputs (geographical variation). **a**–**c** The three measures of outputs included in the model such as the number of discharged patients (Discharged Patients), inpatient days (Inpatient Days), and emergency room treatments (Emergency Room Treatments), respectively

Table 1 contains the descriptive statistics of the variables used in the production set. Table 2 specifies the outputs, inputs and the exogenous factor combinations in the empirical models. Hospitals in the southern regions have, on average, a lower number of personnel (both physicians and nurses) and beds available than those in the northern regions. Hospitals located in northern regions also have, on average, a higher number of discharged patients and emergency room treatments, as well as a higher number of inpatient days, compared to the hospitals in central and southern regions. See Figs. 1 and 2 for a graphical representation of the inputs and outputs at regional level, respectively.

**Table 1:** Descriptive statistics by geographical areas

	North-West	North-East	Centre	South	Italy
Inpatient days	91237.74 (75356.82)	122713.24 (114511.85)	82090.06 (100979.22)	61708.61 (66689.68)	83281.79 (90703.36)
Discharged patients	987.01 (1045.32)	986.72 (1556.95)	776.88 (986.64)	644.70 (760.09)	799.84 (1060.71)
Emergency room treatments	1520.97 (2490.24)	1627.07 (4317.33)	1222.27 (1959.88)	963.50 (1598.89)	1247.37 (2549.33)
Physicians	181.75 (147.46)	214.43 (198.03)	189.52 (221.03)	152.93 (156.71)	178.82 (183.55)
Nurses	376.86 (325.51)	589.51 (537.20)	418.73 (463.49)	324.65 (311.18)	407.18 (416.43)
Other	406.94 (338.22)	497.62 (463.43)	309.70 (428.67)	222.41 (240.64)	327.02 (374.12)
Beds	37.22 (48.92)	24.87 (29.71)	24.89 (39.90)	18.52 (20.66)	24.50 (34.43)
MRI scans	0.0148 (0.004)	0.0154 (0.004)	0.0172 (0.0106)	0.0119 (0.0086)	0.0144 (0.0084)
Public	0.51 (0.50)	0.89 (0.31)	0.84 (0.37)	0.92 (0.27)	0.83 (0.38)
Private	0.49 (0.50)	0.11 (0.31)	0.16 (0.37)	0.08 (0.27)	0.17 (0.38)
Recovery plans	0.50 (0.50)	0.00 (0.00)	0.48 (0.50)	0.87 (0.34)	0.54 (0.50)
Observations	134	148	216	308	806

Authors’ elaboration

**Table 2:** Estimating hospitals’ efficiency—specification of outputs and inputs and exogenous factors

Variables	Definition
Inputs	
Physicians	# of physicians
Nurses	# of nurses
Other	# of other personnel
Beds	# of available beds
MRI scans	# of Magnetic resonance imaging/thousand population (province level)
Outputs	
Discharged patients	# of discharged patients
Inpatient days	# of inpatient days
Emergency room treatments	# of emergency room treatments
Explaining the inefficiency	
Private	Dummy variable taking the value of 1 if the hospital has a private or accredited private health care ownership, and 0 otherwise
Recovery plans	Dummy variable taking the value of 1 if the hospital is located in a region included in the recovery plan, and 0 otherwise
North	Dummy variable taking the value of 1 if the hospital is located in a region located in the North, and 0 otherwise

Authors’ elaboration

### Composite index of efficiency: benefit of doubt approach

Hospitals exhibit a wide variation in the quality of the product and its characteristics being particularly relevant on the output sides. To control for these differences, we construct a composite index consisting of the three output variables described above. The main aim is to avoid common subjectivity on the weight selection by proposing an endogenous weighting mechanism. Therefore, variables in which hospitals have a comparative advantage are more heavily weighted than those in which hospitals have a lower comparative advantage, or even a comparative disadvantage. Since the strengths of hospitals differ, the weights on the performances should differ as well. For example, hospital X may perform relatively poorly with regard to the number of discharged patients. Therefore, in an endogenous weighting, the output ‘number of discharged patients’ will be assigned a lower weight. In contrast, outputs where the hospitals perform relatively well, for instance, the number of emergency room treatments, will obtain a higher weight. In a similar model the weights are observation-specific, diverging from the previous literature and practices. The idea corresponds to the BoD model, a concept that was first developed by Melyn and Moesen (1991).^15^ Using BoD, each hospital gains its own weights that maximize (or minimize) the impact of the criteria in which the hospital performs relatively well (or poorly) compared to others.^16^ The BoD scores are used as composite output to measure the efficiency of hospitals.

## Results

This section is divided into two sub-sections. First, we examine the results of the hospitals’ metafrontier production function based on a stochastic frontier framework (§4.1). The results of the analysis of the inequality in hospital efficiency are then presented and discussed (§4.2).

### The hospitals’ metafrontier production function

Following the approach suggested by Huang et al. (2014), we apply the likelihood ratio test for the null hypothesis that the production frontiers are the same for the two groups of hospitals (those operating in 2007 and 2016) and find that the null hypothesis is rejected supporting the idea that the sample hospitals are operating, over a distance of almost a decade, using heterogeneous technologies. Therefore, the existence of a potential production technology gap justifies the estimation of the metafrontier production function in hospital performance/efficiency.

Table 3 reports the hospitals’ stochastic frontier estimates. Columns 1 and 2 report the estimates of the hospital group-specific stochastic frontiers for both years 2007 and 2016, respectively. The ratio of the group-specific production frontier to the metafrontier is reported in Column 3. All input variables have a positive and statistically significant effect on the outcomes of the hospitals (BoD composite index of efficiency). Being located in a region which is included in the recovery plan is negatively correlated with the technical level of inefficiency. This evidence suggests that being included in such plans not only helps to contain costs, but also seems to have positive consequences on the efficiency of the hospitals (Bordignon et al., 2020, Di Novi et al., 2019). Private hospitals outperform public hospitals. Finally, operating in more economically developed areas (North area of the country) is associated, on average, with higher efficiency. Given the gap in economic development between northern and southern Italy, this result raises serious social issues under the equity profile. Indeed, all else equal, hospitals that operate in southern Italy are required to provide extra effort to produce the same level of output. To deal with multiple outputs in the stochastic frontier framework, we also use as robustness analysis a distance function approach, still assuming a translog functional form. The results, reported in Table 7 in the Appendix, corroborate our results implying that the functional form does not invalidate the findings of the analysis.^17^

**Table 3:** The hospital group stochastic frontier estimates

	(1)	(2)	(3)
Y = Output BOD	1st step: Year 2007	1st step: Year 2016	2nd step
	TE	TE	TGR
ln(Physicians)	0.296*** (0.0902)	0.318*** (0.0710)	0.308*** (0.0119)
ln(Nurses)	0.394*** (0.110)	0.449*** (0.0795)	0.485*** (0.0114)
ln(Beds)	0.0344 (0.0212)	0.113*** (0.0210)	0.0787*** (0.00348)
ln(MRI scans)	0.0939*** (0.0289)	−0.0167 (0.0291)	0.0168*** (0.00354)
ln(Physicians)^2^	0.105 (0.122)	−0.281** (0.141)	0.249*** (0.0221)
ln(Nurses)^2^	−0.167 (0.197)	−0.270 (0.171)	0.188*** (0.0292)
ln(Beds)^2^	−0.00703 (0.0343)	0.0562** (0.0250)	0.0717*** (0.00583)
ln(MRI scans)^2^	0.0145 (0.0239)	0.00782 (0.0226)	0.00994*** (0.00246)
ln(Physicians)*ln(Nurses)	0.614** (0.253)	1.049*** (0.307)	0.0252 (0.0561)
ln(Physicians)*ln(Beds)	−0.0814 (0.151)	−0.221* (0.118)	−0.164*** (0.0284)
ln(Physicians)*ln(MRI scans)	−0.0878 (0.219)	0.00672 (0.141)	0.0357 (0.0305)
ln(Nurses)*ln(Beds)	−0.0947 (0.162)	0.294*** (0.100)	−0.0932*** (0.0214)
ln(Nurses)*ln(MRI scans)	−0.199 (0.238)	−0.365*** (0.118)	−0.0151 (0.0226)
ln(Beds)*ln(MRI scans)	0.0834 (0.0528)	−0.0349 (0.0455)	−0.0155** (0.00770)
North (ref. group: Centre-South)	−0.160 (0.107)	0.0365 (0.0700)	−0.196*** (0.00967)
Constant	0.354*** (0.0697)	0.295*** (0.0498)	0.287*** (0.00642)
Variance of inefficiency component			
Recovery plans	−3.192 (2.870)	−0.945** (0.411)	0.547** (0.247)
Private (ref. group public)	−1.365*** (0.264)	−0.941*** (0.224)	−5.129*** (0.175)
North (ref. group Centre-South)	−4.775* (2.666)	−1.331** (0.529)	−4.591*** (0.319)
Variance of stochastic component			
Constant	−2.021*** (0.135)	−3.067*** (0.249)	−5.340*** (0.162)
Observations	403	403	806

Standard errors, clustered at regional level, in brackets

*p < 0.10, **p < 0.05, ***p < 0.01

**Fig. 3:**
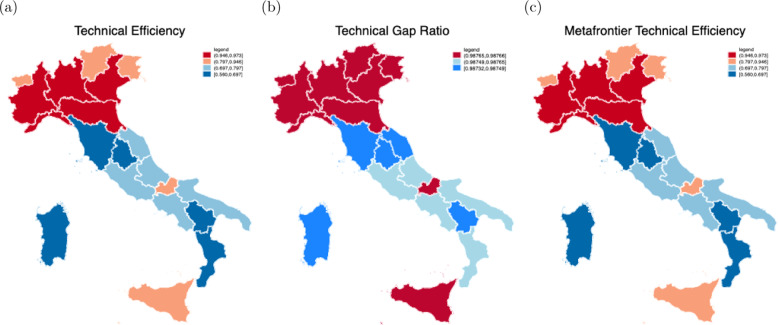
Hospitals’ efficiency scores (geographical variation). **a**–**c** The measures of hospitals’ efficiency scores such as Technical Efficiency, Technical Gap Ratio, and Metafrontier Technical Efficiency, respectively

Table 4 reports the summary statistics of various efficiency scores, at regional level, for the two groups of regions (in 2007 and 2016)^18^ while Fig. 3 reports a graphical representation of the hospitals’ efficiency scores at regional level. All regions in 2016 (with the exception of Lazio, Puglia, Sardegna, Sicilia and Toscana) seem to be more technically efficient (average TE = 0.7839) with respect to their own peer group in 2007 (average TE = 0.7781). Hospitals operating in 2016 seem to be highly more efficient (average TGR = 0.9876) compared to those in 2007 (average TGR = 0.9630) in adopting the best available hospital-operating technology as measured in the technology gap ratio. The hospitals in 2007 are less technically efficient (average MTE = 0.7614) with respect to the same hospitals in 2016 (average MTE of 0.7838) as measured by the metafrontier technical efficiency. Overall, on average the TGR scores play a more important role in the determination of the ranking in MTE, suggesting that sources of inefficiency for hospitals operating in 2007 come especially from the technology used rather than from managerial inefficiency.

**Table 4:** The estimates of the hospitals’ metafrontier

	Year 2007			Year 2016			Overall		
Regions	TE	TGR	MTE	TE	TGR	MTE	TE	TGR	MTE
Abruzzo	0.6868	0.9555	0.6643	0.7120	0.9876	0.7120	0.6994	0.9716	0.6881
Basilicata	0.6602	0.9403	0.6287	0.6714	0.9873	0.6711	0.6658	0.9638	0.6499
Calabria	0.5863	0.9550	0.5665	0.6051	0.9876	0.6050	0.5957	0.9713	0.5858
Campania	0.6822	0.9559	0.6611	0.7127	0.9876	0.7127	0.6974	0.9718	0.6869
Emilia Romagna	0.8917	0.9877	0.8917	0.9476	0.9877	0.9476	0.9203	0.9877	0.9203
Friuli Venezia Giulia	0.8634	0.9877	0.8634	0.9398	0.9877	0.9398	0.9016	0.9877	0.9016
Lazio	0.7678	0.9656	0.7520	0.7225	0.9877	0.7225	0.7451	0.9766	0.7372
Liguria	0.9506	0.9877	0.9506	0.9573	0.9877	0.9573	0.9540	0.9877	0.9540
Lombardia	0.9716	0.9877	0.9716	0.9731	0.9877	0.9731	0.9724	0.9877	0.9724
Marche	0.7248	0.9210	0.6766	0.7471	0.9873	0.7469	0.7360	0.9542	0.7117
Molise	0.7825	0.9608	0.7621	0.8480	0.9877	0.8479	0.8152	0.9742	0.8050
P.A. Bolzano	0.8796	0.9877	0.8796	0.9436	0.9877	0.9436	0.9116	0.9877	0.9116
P.A. Trento	0.8917	0.9877	0.8917	0.9479	0.9877	0.9479	0.9198	0.9877	0.9198
Piemonte	0.9341	0.9877	0.9341	0.9673	0.9877	0.9673	0.9507	0.9877	0.9507
Puglia	0.7522	0.9592	0.7312	0.7430	0.9876	0.7430	0.7476	0.9734	0.7371
Sardegna	0.6653	0.9543	0.6425	0.5606	0.9873	0.5604	0.6129	0.9708	0.6014
Sicilia	0.8926	0.9769	0.8830	0.8815	0.9877	0.8815	0.8871	0.9823	0.8822
Toscana	0.6599	0.9218	0.6157	0.6511	0.9874	0.6509	0.6555	0.9546	0.6333
Umbria	0.6479	0.9032	0.5965	0.6830	0.9873	0.6828	0.6655	0.9453	0.6396
Veneto	0.9109	0.9877	0.9109	0.9533	0.9877	0.9533	0.9321	0.9877	0.9321
Total	0.7781	0.9630	0.7614	0.7839	0.9876	0.7838	0.7810	0.9753	0.7726

Valle d’Aosta is excluded having only one hospitals observation

An interesting heterogeneity is present when single regions are taken into account. Indeed, the TE scores play a more important role in the determination of the lowering (or not notable increasing) MTE ranking in 2016 with respect to 2007 for Lazio, Puglia, Sardegna, Sicilia and Toscana, suggesting that the primary source of inefficiency come from (lower) managerial efficiency rather than the technology undertaken. On the other hand, the TE scores play a more important role in the determination of the ranking in MTE for Emilia-Romagna, Friuli-Venezia Giulia, Liguria, Lombardia, Trentino (P.A. Bolzano and P.A. Trento), Piemonte and Veneto, suggesting that the primary source of efficiency in 2016 comes from (higher) managerial efficiency rather than the technology undertaken (that remain quite constant, although very high). Finally, although Basilicata, Calabria, Marche and Umbria in year 2016 outperform year 2007, the TGR scores play a more important role in determining the ranking in MTE, suggesting that for hospitals located in those regions the main source of efficiency come especially from the technology undertaken rather than managerial efficiency. The opposite is true, instead, for Molise for which the main source of efficiency for hospitals operating in 2016 seems to come from managerial efficiency rather than the technology used. Finally, both TE and TGR scores play an important role in determining the increasing ranking in MTE for Abruzzo and Campania, suggesting that sources of efficiency in 2016 are both (higher) managerial efficiency and the technology undertaken.

### Rising Inequality in Hospital Efficiencies

**Fig. 4:**
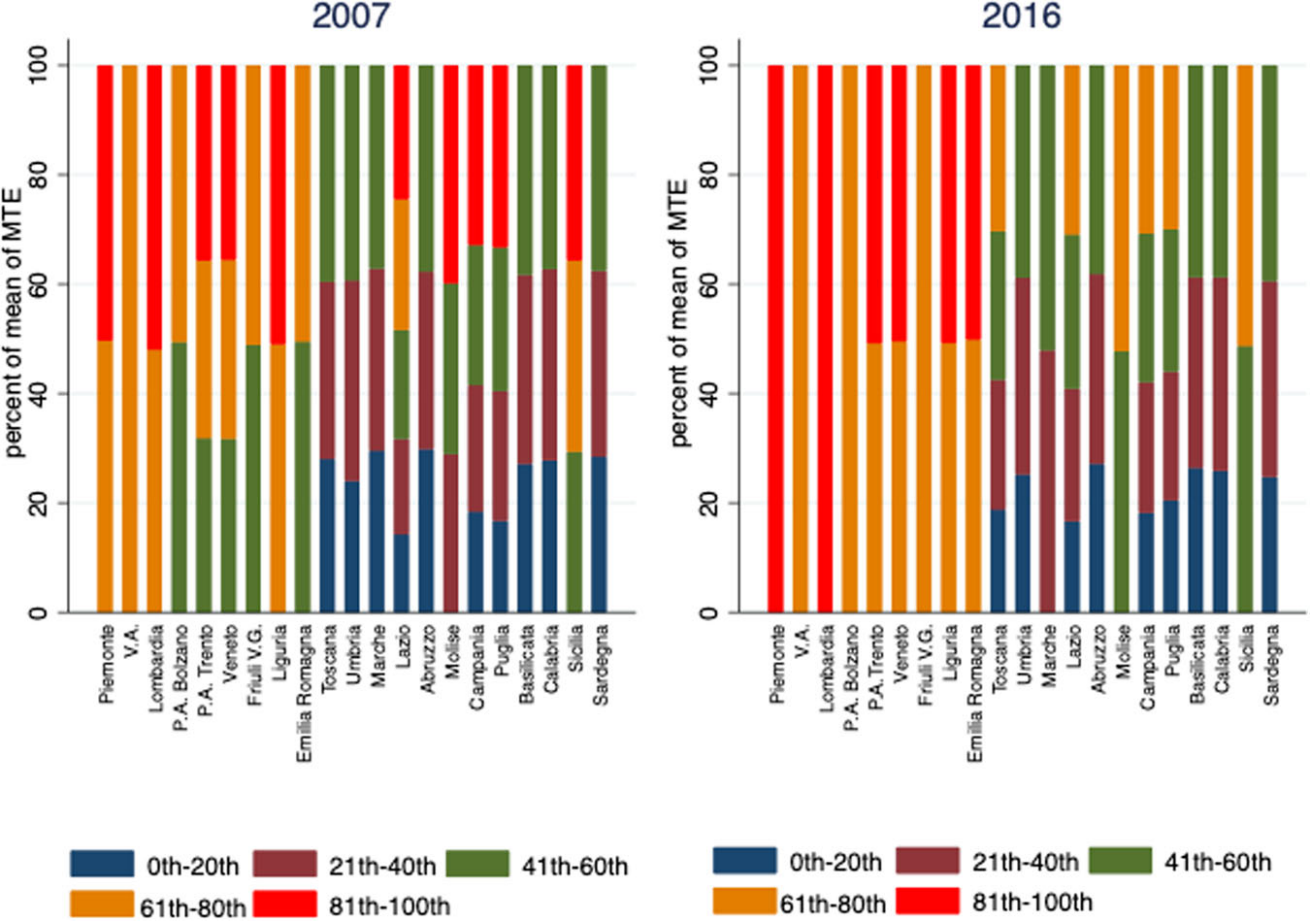
Rank distribution of the hospitals’ efficiency scores at regional level for years 2007 and 2016

To analyze the distribution over time of the ‘more efficient and ‘less efficient’ hospitals, we divide the rank distribution of the hospitals’ scores into 5 percentiles. The graphical representation of the rank distribution is represented in Fig. 4 (the blue, brown, green, orange and red lines illustrate, for each region and for years 2007 and 2016, the share of hospitals that falls in the 20th, 40th, 60th, 80th and 100th rank percentile) and shows a clear and persistent North-South gap in the efficiency performances of hospitals. Indeed, in all the northern regions (Piemonte, Valle d’Aosta, Liguria, Lombardia, Trentino-Alto Adige, Veneto, Friuli-Venezia Giulia, Emilia-Romagna) the hospitals’ efficiency scores reached the highest 61th–80th and 81th–100th percentiles. The gap between the central-northern and southern regions did not change substantially between 2007 and 2016. A clear pattern emerges: none of the southern hospitals reach the highest percentile both in 2007 and 2016. More specifically, in the southern regions and in the two periods analyzed, the distribution of efficiency never reaches the highest percentiles in Basilicata, Calabria and Sardegna.

In line with the previous literature, the findings highlight that in the last decades no process of convergence between the southern regions and those in the north has been observed (Lagravinese et al., 2019). The neglect inefficiencies are still evident between the two macro areas (north-south) and in 2016 the best hospital in the south never reaches the results of the northern hospitals. This result may explain how health mobility between northern and southern regions has increased in recent years, posing serious problems of inequality due to the fact that only individuals with higher incomes can afford to be treated outside their own region (Balia et al., 2018, 2020).

The rank distribution can then be further decomposed to estimate the variability between and within regions according to Eq. (10). To this purpose, Table 5 reports the Theil index for hospital’ performances, with two components: the between regions (estimated within 20 regions) and the within regions. Table 5 shows that between 2007 and 2016, the total inequality of the country increased from 0.022 to 0.025, suggesting that the differences between areas of the country have remained almost unchanged. Much of this inequality is determined by between inequality (around 63.9% of the total). In both years 2007 and 2016, the between inequality increased from 0.014 to 0.015 and the within inequality slightly increased from 0.008 to 0.009.

**Table 5:** Theil index: total, between and within inequality

Year	Total	Between Inequality	Within Inequality
2007	0.0227	0.0146	0.0081
2016	0.0253	0.0155	0.0099

Authors’ elaboration

Additionally, we also calculate the Theil index for each region to further examine how the within inequality has changed in the single regions. The results, summarized in Table 6, show that in general, as was to be expected, inequality in the regions is not as high. This is explained by the fact that by now the regions have outlined the distribution of the service on the local territory where all the hospitals follow guidelines and homogeneous protocols in the regional territory. However, some differences can be seen. In the central-northern regions (net of the regions of Umbria and especially Lazio), it is generally observed that the differences in the same region are minimal with values very close to zero, while the differences are greater among the hospitals in the southern regions. Between 2007 and 2016, an increase in the within inequality occurred especially in Calabria and Sardegna.

**Table 6:** Theil Index Within inequality by regions

Regions	Within Inequality Year 2007	Within Inequality Year 2016
Abruzzo	0.005131	0.010803
Basilicata	0.007533	0.015858
Calabria	0.009963	0.028853
Campania	0.02072	0.015163
Emilia Romagna	0.000024	0.000002
Friuli Venezia Giulia	0.000405	0.000016
Lazio	0.021015	0.023860
Liguria	0.000366	0.000113
Lombardia	0.000097	0.000010
Marche	0.003351	0.001524
Molise	0.006414	0.001200
P.A. Bolzano	0.000070	0.000003
P.A. Trento	0.000637	0.000055
Piemonte	0.000100	0.000008
Puglia	0.011460	0.007749
Sardegna	0.008694	0.038003
Sicilia	0.002874	0.000411
Toscana	0.010360	0.013241
Umbria	0.023173	0.020257
Veneto	0.000697	0.000065

Authors’ elaboration.

Valle d’Aosta is excluded havingonly one hospitals observation

## Concluding remarks and policy implications

### Conclusion

This paper investigates the efficiency of Italian hospitals and how their performances have changed over the period 2007–2016. We apply the BoD approach to determine a composite index that considers the multi-dimensionality of the hospital outcome to be used as main output in a metafrontier production function based on a stochastic frontier framework. This procedure disentangles the group-specific frontiers and the metafrontier, further decomposing the efficiency scores of various groups of hospitals into technical efficiency scores and technology gaps.

The main findings show that almost all regions in 2016 (with some exceptions) seem to be more technically efficient than their own peer group in 2007, being, on average, also more efficient in adopting the best available hospital-operating technology. More heterogeneity is found when looking at the single regions. Indeed, managerial efficiency rather than the efficiency in adopting the best available technology seems to be more important (or not important) in determining the lowering (increasing) in the metafrontier technical efficiency ranking, especially for hospitals located in southern regions. Furthermore, with a closer look at the distribution of the efficiency scores across time, the empirical evidence shows a clear and persistent North-South gap in the efficiency performances of hospitals that did not change substantially between 2007 and 2016. Finally, when we further decomposed the rank distribution in order to estimate the variability between and within regions, the results show an increase in the inequality in terms of efficiency between the areas of the country mostly determined by between region inequality. An increase in within region inequality is also found, especially for the southern regions.

A possible shortcoming of our empirical strategy is worthy to be further discussed. We apply the BoD methodology using the typical construction of the meta-frontier which is estimated under the assumption that the metaset is convex. Violating this assumption could make the estimates biased (see Kerstens et al. (2019) for a discussion). The convexity assumption is usually justified using a time divisibility argument (Hackman, 2008, Shephard, 1970) according to which if production processes are time divisible, then a (hospital) manager could use, in some cases, an input to produce a specific output, and then use a second input to produce a further specific output the rest of the time. Our knowledge of hospital organization leads us to believe that this is not the case for the groups of hospitals operating in 2007 and 2016 suggesting that the technology-specific production possibilities sets may not be convex (i.e., possible differences in the methods that are available to transform inputs into outputs or in production environments).

Finally, given the data constraints and unavailability, more dimensions of inputs and outputs could not be used in the empirical analysis. However, besides labour and capital (represented by number of beds), the consumable (e.g., supplies in medical, drugs, and surgical, etc.) is also an important aspect to be accounted in as the inputs (see, for an example, Chowdhury and Zelenyuk (2016)). Moreover, the number of employees is used as the inputs for labour, even though the full-time equivalent staff is also widely accepted as an important variable in hospital efficiency analysis (see for comprehensive reviews Hollingsworth (2008), O’Neill et al. (2008), Worthington (2004)). These more thorough variables should be taken into account for empirical models and studies in the future.

### Policy implications

Several implications can also be derived from our analysis.

The findings highlight the still unresolved social-economic dualism between the northern and southern regions of the country. Indeed, a persistent territorial divide in the regional health care in Italy has been seen to be, at least partially, the possible cause of a large heterogeneity of mortality rates among regions (Arcà et al., 2020, Lagravinese et al., 2019). We find that the gap between the central-northern and southern regions not only did not change substantially between 2007 and 2016, but also that none of the central-southern hospitals reach the highest percentile in both periods, confirming that in the last decades no process of convergence between the southern regions and those in the north has been observed.

Although, there is evidence of the general positive effect of decentralization on health performance in Italy (Atella et al., 2019, Bordignon et al., 2020, Di Novi et al., 2019), our study, along with some recent literature (Arcà et al., 2020, Depalo, 2019, Lagravinese et al., 2019), provides evidence that such improvements may not involve all the health dimensions and all the regions, and, more importantly, may not solve, even partially, the increasing gap between northern and southern regions. The decentralization process therefore does not seem to have helped bridging the gap between hospitals in the north and those in the south. Indeed, the differences have also (albeit slightly) worsened over the years. Certainly, further analyses are needed in the future to more carefully indicate whether the efficiency changes are the result of such policies in filling up the gap, given that also other social-economic factors changed in the study period (e.g., the demography changes in different regions). However, in the next few years, also in light of the critical issues on regional organization revealed by the recent pandemic due to the COVID-19 virus, national policies will have to be implemented to bridge the infrastructural and economic gap in the southern regions.

## Appendix

Table 7

**Table 7:** The hospital group stochastic frontier estimates

	(1)	(2)	(3)
Y = ln(Impatient Days)	1st step: Year 2007	1st step: Year 2016	2nd step
	TE	TE	TGR
ln(Physicians)	0.304*** (0.0737)	0.271*** (0.0589)	0.293*** (0.0136)
ln(Nurses)	0.376*** (0.0820)	0.294*** (0.0610)	0.344*** (0.0154)
ln(Beds)	0.260*** (0.0356)	0.354*** (0.0321)	0.260*** (0.00690)
ln(MRI scans)	−0.00670 (0.0238)	−0.0488 (0.0256)	0.00549 (0.00472)
ln(Discharged Patients)	−0.311*** (0.0333)	−0.379*** (0.0365)	−0.330*** (0.00661)
ln(Emergency Room Treatments)	0.00145 (0.0186)	0.0282* (0.0157)	0.0289*** (0.00360)
ln(Physicians)^2^	0.0800 (0.112)	−0.257** (0.123)	−0.0296 (0.0391)
ln(Nurses)^2^	0.0529 (0.194)	−0.349** (0.138)	−0.125*** (0.0428)
ln(Beds)^2^	−0.0473 (0.0546)	−0.0322 (0.0381)	−0.0216 (0.0133)
ln(MRI scans)^2^	−0.0185 (0.0266)	−0.00111 (0.0179)	0.000752 (0.00515)
ln(Physicians)*ln(Nurses)	0.155 (0.234)	0.693*** (0.254)	0.429*** (0.0783)
ln(Physicians)*ln(Beds)	−0.393** (0.167)	−0.305** (0.127)	−0.280*** (0.0358)
ln(Physicians)*ln(MRI scans)	−0.0304 (0.204)	−0.00243 (0.150)	−0.0746** (0.0359)
ln(Nurses)*ln(Beds)	0.261 (0.168)	0.479*** (0.123)	0.310*** (0.0337)
ln(Nurses)*ln(MRI scans)	−0.0998 (0.196)	−0.174 (0.118)	−0.125*** (0.0324)
ln(Beds)*ln(MRI scans)	0.274*** (0.0788)	0.0206 (0.0593)	0.161*** (0.0195)
ln(Discharged Patients)^2^	−0.126*** (0.0287)	−0.159*** (0.0232)	−0.141*** (0.0110)
ln(Emergency Room Treatments)^2^	0.00168 (0.00884)	0.00825 (0.0122)	0.0110*** (0.00197)
ln(Discharged Patients)*ln(Physicians)	0.160* (0.0866)	0.160** (0.0679)	0.135*** (0.0178)
ln(Discharged Patients)*ln(Nurses)	−0.149* (0.0848)	−0.233*** (0.0550)	−0.165*** (0.0179)
ln(Discharged Patients)*ln(Beds)	0.0589* (0.0346)	0.0658** (0.0322)	0.0608*** (0.0111)
ln(Discharged Patients)*ln(MRI scans)	−0.147*** (0.0334)	−0.0341 (0.0387)	−0.0951*** (0.00870)
ln(Emergency Room Treatments)*ln(Physicians)	−0.0210 (0.0338)	−0.0651 (0.0547)	−0.0409*** (0.00796)
ln(Emergency Room Treatments)*ln(Nurses)	0.0639* (0.0358)	0.0961** (0.0376)	0.0704*** (0.00779)
ln(Emergency Room Treatments)*ln(Beds)	0.0103 (0.0120)	0.0150 (0.0139)	0.00604** (0.00267)
ln(Emergency Room Treatments)*ln(MRI scans)	0.00907 (0.0152)	0.00824 (0.0206)	0.00233 (0.00364)
North (ref. group: Centre-South)	−0.206*** (0.0593)	−0.0123 (0.0415)	−0.197*** (0.0118)
Constant	0.393*** (0.0625)	0.470*** (0.0482)	0.477*** (0.00977)
Variance of inefficiency component			
Recovery plans	−2.617* (1.374)	−0.620** (0.270)	−1.197* (0.629)
Private (ref. group public)	−1.477*** (0.229)	−1.045*** (0.181)	−4.711*** (0.257)
North (ref. group Centre-South)	−31.95*** (2.557)	−1.630*** (0.284)	−34.73*** (3.380)
Variance of stochastic component			
Constant	−2.445*** (0.119)	−3.838*** (0.279)	−4.852*** (0.0664)
Observations	403	403	806

Standard errors, clustered at regional level, in brackets

**p* < 0.10, ***p* < 0.05, ****p* < 0.01

## Footnotes

1 LEA is a list of health care services that the central government requires to be guaranteed in all regions, being recently updated by a ministerial decree in 2017.

2 Some regions were subject to specific recovery plans, called “Piani di Rientro”, implemented to recover from budget deficit related to health expenditures.

3 To address shortcomings associated with multiple outputs and avoid limitations arising from the use of simple arithmetic or geometric averages, a few solutions have been proposed such as the multi-directional efficiency analysis approach (Tziogkidis et al., 2020). With regard to an application in the health sector, Castelli et al. (2015) impose an explicit set of weights with which to combine diverse outputs into a single index.

4 For this reason, decision makers should not be worried about unfair weighting schemes since each hospital is put in its most favorable condition, since any other weighting scheme would generate a lower composite index (Cherchye et al., 2007).

5 The use of a single composite outcome allows us to take into account the complexity of the hospitals in the production function, avoiding a limit of the Stochastic Frontier Analysis, for example, the use of a single output for each production function.

6 It relies on the general regularity properties such as free disposability, convexity, and assumptions concerning the returns to scale and it does not require any assumption on the error term either, because it assumes that any deviation from frontier is due to inefficiency.

7 It requires an ex-ante assumption of a functional form for the underlying production function (that may however violate the monotonicity, concavity/convexity and homogeneity conditions).

8 Alternative estimation methods of stochastic frontier models are also available such as the local likelihood estimation method introduced by Kumbhakar et al. (2007) and the extended methods based on it, such as Park et al. (2015), allowing for categorical variables in the local likelihood method, and Simar et al. (2017), using local least squares method instead of the local likelihood method.

9 Due to the fact that the second step estimation is a linear programming algebraic calculation, the resulting metafrontier has no statistical properties. Moreover, the characteristic of the environment in which the unit analyzed operate cannot be incorporated.

10 Indeed, it applies the conventional maximum likelihood method to estimating the parameters of the SMF regression.

11 A commonly used indicator of medical technology; see, for instance, Retzlaff-Roberts et al. (2004) and Afonso and St Aubyn (2006).

12 The linear homogeneity is ensured by dividing all inputs by one input (in our case the number of other personnel) and symmetry conditions are also imposed.

13 Plans were signed in February 2007. To date, three waves of Piani di Rientro have been concluded. The first round of plans formally ended in 2009; afterwards, regions were allowed to sign new plans over the periods of 2010–2012 and 2013–2015. Ten regions underwent the program: Lazio was the first in February 2007; Abruzzo, Campania, Liguria, Molise and Sardegna joined the plan in March of the same year; Sicilia joined the plan in July of the same year. Of these regions, Liguria met requirements at the end of the first round (in 2010 instead of 2009) and left the program, whereas all of the other regions signed a new plan for 2010–12. For the second round, Calabria also signed a plan in December 2009 and Piemonte and Puglia signed a plan in 2010.

14 See Depalo (2019) and Arcà et al. (2020) for an estimation of the causal effects on health-related outcomes of recovery plans as well as Bordignon et al. (2020) for an evaluation of the recovery plans in terms of cost containment and safeguarding of an acceptable provision of health care services.

15 See De Witte and Hudrlikova (2013), De Witte and Rogge (2011) and Coco et al. (2020) for an application of this method to higher education.

16 Therefore, for each hospital, the BoD procedure searches for weights that maximize the impact of the strengths and minimize the influence of the relative weaknesses. This means that in absence of any other detailed information on the true weights, the BoD procedure assumes that representative weights can be inferred from looking at the relative strengths and weaknesses.

17 We use the number of inpatient days for the normalization of the outcome variable (O’Donnell and Coelli, 2005). Although the choice of the normalizing the outcome variable is innocuous when using stochastic frontier models as they are mathematically equivalent, for robustness, we also conduct a sensitivity analysis normalizing by the number of discharged patients and by the number of emergency room treatments (results on the different normalization are similar and available on request).

18 The efficiency scores are obtained using data at individual hospital level and then summed up at regional level. The individual hospital efficiency scores are available on request.
